# Polymeric approach to combat drug-resistant methicillin-resistant *Staphylococcus aureus*

**DOI:** 10.1007/s10853-021-05776-7

**Published:** 2021-01-25

**Authors:** Shreya Kanth, Akshatha Nagaraja, Yashoda Malgar Puttaiahgowda

**Affiliations:** Department of Chemistry, Manipal Institute of Technology, Manipal Academy of Higher Education, Manipal, 576104 India

## Abstract

**Abstract:**

The current global death rate has threatened humans due to increase in deadly unknown infections caused by pathogenic microorganisms. On the contrary, the emergence of multidrug-resistant bacteria is also increasing which is leading to elevated lethality rate worldwide. Development of drug-resistant bacteria has become one of the daunting global challenges due to failure in approaching to combat against them. Methicillin-resistant *Staphylococcus aureus* (MRSA) is one of those drug-resistant bacteria which has led to increase in global mortality rate causing various lethal infections. Polymer synthesis can be one of the significant approaches to combat MRSA by fabricating polymeric coatings to prevent the spread of infections. This review provides last decade information in the development of various polymers against MRSA.

**Graphical abstract:**

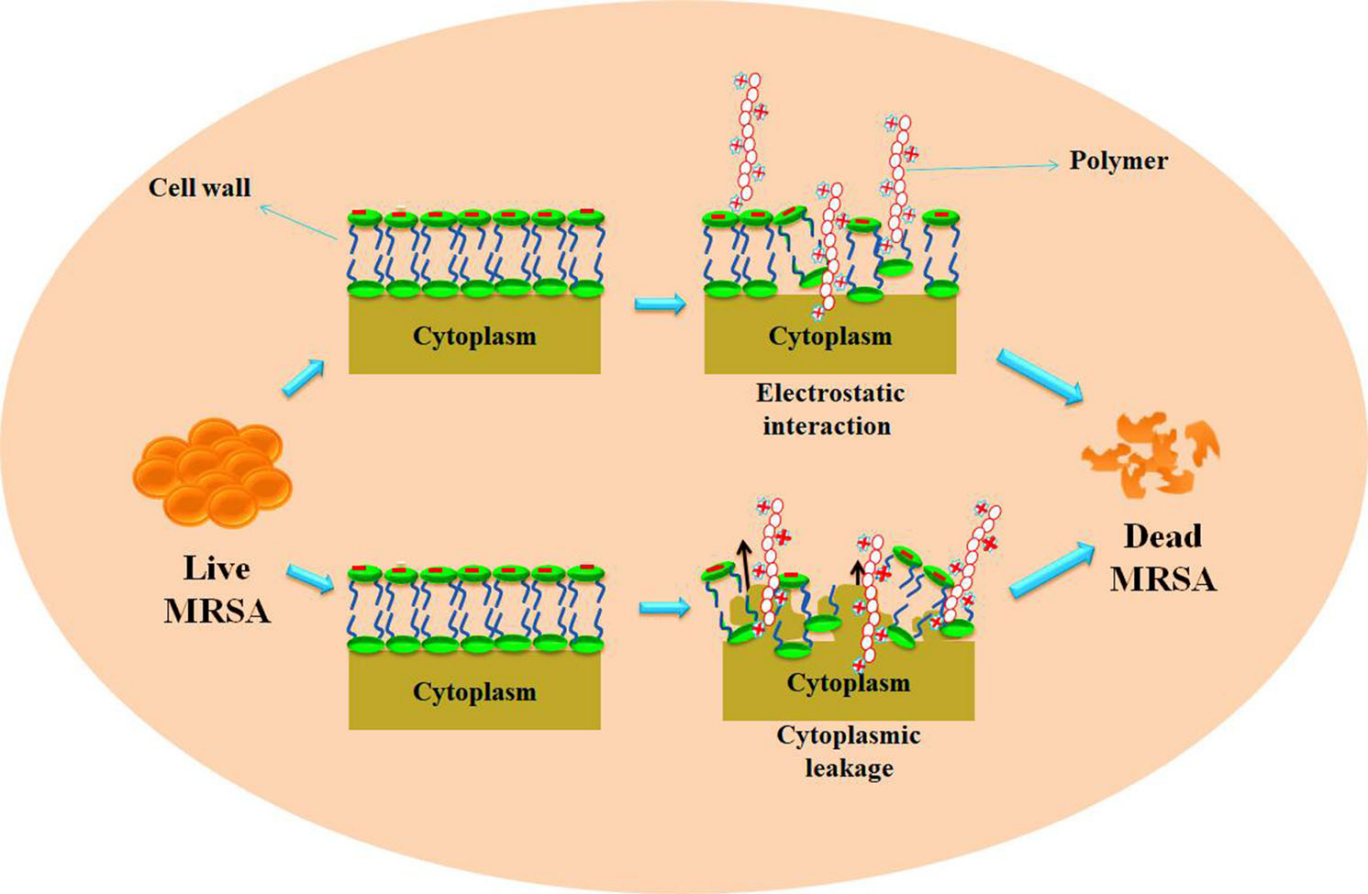

## Introduction

Throughout recent years, biofilm formation and the advent of drug resistance microbes to modern antibiotics have been a significant problem in the area of biomedical science [1–6]. One-quarter of deaths occur worldwide due to microbial infections, particularly in developing countries [7–10]. As per the data reported by US Centers for Disease Control and Prevention, millions of people are infected with antibiotic-resistant bacteria [11, 12]. Since 1980, the number of hospital-acquired infections (HAIs) has increased steadily worldwide, due to rise and spread of multidrug-resistant (MDR) bacteria [13]. MDR is defined as the resistance or insensitivity of microorganisms to the antimicrobial drugs enforced [14]. MDR is recognized as the major cause for the emergence of public health problems [15]. The microorganisms which are resistant to drugs are termed as ESKAPE pathogens [16]. These ESKAPE pathogens comprise both gram-positive and gram-negative species (*Enterococcus faecium*, *Staphylococcus aureus*, *Klebsiella pneumoniae*, *Acinetobacter baumannii*, *Pseudomonas aeruginosa* and* Enterobacter* species), and these species are responsible for the spread of nosocomial infections and HAIs worldwide [17, 18]. Few most important pathogens which cause HAIs are vancomycin-resistant *Enterococcus* spp. (VRE), *Clostridium difficile*, *A. baumannii*, methicillin-resistant *S. aureus* (MRSA), *P. aeruginosa* and Enterobacteriaceae strains [13, 19–22]. Pathogens such as Candida species, viruses [influenza, hepatitis B viruses, adenoviruses, parainfluenza, noroviruses, rotaviruses and severe acute respiratory syndrome (SARS)-associated coronaviruses] also survive on medical equipments and surfaces, to a lesser extent [23, 24].

Many studies have found that > 60% of HAI's around the world is due to the attachment of different pathogens on fracture fixation devices, medical devices and implants and urinary catheters [13, 25–30]. Therefore, there is a necessity to fight against the spreading of MDR.

Among these pathogens, numerous infectious diseases, namely purulent meningitis, pleuritis, pneumonia, tympanitis and bloodstream infections, are caused by gram-positive methicillin-resistant *S. aureus* (MRSA) [31, 32]. Methicillin-resistant *S. aureus *(MRSA) pathogen is a major cause for nosocomial and community infections throughout the world [33]. It causes severe infections which exhibit notable antibiotic resistance [34, 35]. Infections of the skin caused by invasive pathogens begin when the outer skin is affected due to skin diseases, like atopic dermatitis and injury, such as a burn, and these invasive skin infections are majorly caused by *S. aureus* which includes MRSA [36–39]. Overall mortality rate is leading to 10–30% due to the treatment failure of MRSA-induced bacteraemia [31, 40]. Due to the increased virulence of epidemic strains the restraint of *S. aureus*, particularly MRSA is very difficult [36]. Transmission control is concentrated on the prevention of spread of these pathogens by environment (contaminated equipments and surfaces) and health-care workers (contaminated hands) [41]. A relevant amount of microbes is disrupted by disinfection process, using chemical agents like aldehydes, quaternary ammonium compounds, halogens and alcohols, or heat and radiation [13, 22, 42–46]. Sterilization and disinfection processes and at times aerosols need to be used to clean air to constraint hospital infections [22, 42]. Developing novel antibacterial methods to counter MRSA infections is an urgent need.

In recent years, several polymer-coated antimicrobial surfaces, antimicrobial surfaces and polymer-based antimicrobial hydrogels with several properties are developed to deal with clinical threats [1, 47–52]. Antimicrobial polymers are strong candidates among such antimicrobial materials, because they can successfully kill microbes and help to eliminate such pathogens [31, 53]. Such antimicrobial polymers target primarily the microbial membrane and show less tendency to cause the development of resistance [1]. They are used in environments at high risk of contamination, with uses such as self-sterilizing catheter tubes, medical drug coatings, surgical devices and wound dressings [54, 55]. Thus, synthetic polymers are commonly used as a new molecular framework for the development of antimicrobials that are active against drug-resistant bacteria [56–60]. This article focuses on various synthesized polymers and polymeric coatings used against methicillin-resistant *S. aureus* (MRSA) and their study on mechanism, minimum inhibitory concentration (MIC) or zone of inhibition (ZOI).

## Polymers containing quaternary ammonium compounds

Hydroxypropyltrimethyl ammonium chloride chitosan (HACC) with various degree of substitution (6%, 18% and 44%) of quaternary ammonium was prepared by the reaction of chitosan with glycidyl trimethylammonium chloride by Peng et al. in 2010. Three bacteria responsible for orthopedic implant-related infections, *Staphylococcus epidermidis*, methicillin-resistant *S. aureus* and *S. aureus*, were used to evaluate the antibacterial activity of these synthesized polymers. The cationic group of the synthesized polymers targets the bacterial cell surface which is negatively charged. HACC (18% substitution) showed excellent potential to inhibit bacterial growth and biocompatibility with osteogenic cells. Biocompatibility and cytotoxicity were also tested for these polymers [61–63].

A series of amphiphilic and biodegradable, broad-spectrum antimicrobial polycarbonates were synthesized by Chin et al. in 2013 by metal-free organocatalytic ring-opening polymerization using (MTC-OCH_2_ BnCl) as monomer. The synthesized polycarbonates were further quaternized by post-polymerization quaternization reaction with quaternary ammonium groups of various pendant structure to obtain cationic polymers. The antimicrobial activity of synthesized polycarbonates was tested against *P. aeruginosa*, *Escherichia coli* and *S. aureus* and clinically isolated nosocomial microbes such as VRE, *C. neoformans*, *A. baumannii* and MRSA. Among the synthesized polycarbonates, polymer pbutyl_20 showed excellent antimicrobial activity and selectivity against clinically isolated drug-resistant microbes (VRE, MRSA and carbapenem resistant *A. baumannii*). Increase in alkyl chain length and hydrophobicity of the polymer raises its tendency to attach to the lipid membrane which leads to membrane disruption and results in cell death. The mechanism of action was studied by field emission scanning electron microscopy [64–67].

Biodegradable antimicrobial polycarbonates, pButyl-20 and pButyl_0.5_Benzyl_0.5–20_, containing cationic groups were synthesized by Cheng et al. in 2015. The polycarbonates were synthesized via organocatalytic ring-opening polymerization of benzyl chloride-functionalized cyclic carbonate monomer. The polymers were further quaternized by *N*,*N*-dimethylbutylamine and *N*,*N*-dimethylbutylamine/*N*,*N-*dimethylbenzylamine (1:1 molar ratio) as the cationic groups. These polymers form pores on the plasma membrane which eventually destroys the bacterial cell which was studied by transmission electron microscopy. The authors concluded that the synthesized polymers had a wide range of antimicrobial activity and were used for treating multidrug resistance MRSA infection [40, 64, 68, 69].

Uppu et al. in 2016 synthesized cationic amphiphilic polymers by polymerization of poly-(isobutylene-*alt*-maleic anhydride) with 3-aminopropyldimethylamine followed by quaternization of the tertiary nitrogen of polymerization product, poly(isobutylene-alt-*N*-(*N*′,*N*′-dimethylaminopropylmaleimide). The cyclized hydrophobic side chains attached to the cationic part far from the macromolecule are responsible for the death of bacteria. The obtained polymers showed good antimicrobial activity against *S. aureus*, MRSA, *E. coli* and vancomycin resistant *E. faecium* [70–73].

Li et al. in 2016 synthesized a novel antimicrobial polymer, poly(*N*,*N*-dimethylaminoethylmethacrylate)-block-poly(l-lacticacid)-block-poly(*N*,*N*-dimethylaminoethylmethacrylate) conjugated with poly(ethylene glycol) (D-PLLA-D@PEG) triblock copolymers by the combination of atom transfer radical polymerization. These polymers were further quaternized with two different chemical compositions (D-PLLA-D@Q 1: 12.3%; D-PLLA-D@Q 2: 26.2%). The amphiphilic and cationic groups are present in the polymer targets and rupture the bacterial membrane through electrostatic interaction and infuse into the membrane lipid domains and decrease the resistance of bacteria. All these polymers exhibited good antimicrobial activity against methicillin-resistant *S. aureus.* The authors concluded this could be used for coating hospital surfaces, gowns and prevents MDR bacteria [74–77].

Based on cationic polyaspartamide derivatives, four kinds of novel biodegradable antibacterial polymer with various lengths of side chains were synthesized by using β-benzyl-l-aspartate *N*-carboxy anhydride through ring-opening polymerization by Yan et al. in 2019. The synthesized Q-PAsp (BDA) catiomers showed a wide range of antibacterial activity against gram-negative and gram-positive bacteria. To enhance the biocompatibility of polycations, carboxylatopillar[5]arene (CP[5]A) was introduced to polymeric catiomers. The synthesized polymers attach to the surface of bacteria through electrostatic interaction (between polymer and negatively charged cell membrane) and disturb the normal functioning of bacteria leading to structural destabilization resulting in bacterial cell death. The mechanism was studied by scanning electron microscopy and confocal laser scanning microscopy. The authors concluded Q@CP[5]A exhibited excellent activity against in vivo MRSA and hence could be used for wound healing and inhibit antibiotic-resistant pathogenic bacterium [78–81] (Table 1).

**Table 1:** List of polymers containing quaternary ammonium compounds synthesized by various authors against MRSA and other pathogenic microorganisms

Year	Author	Polymer	Antimicrobial activity	MIC or ZOI	References
2010	Peng et al.		MRSA *S. aureus* *S. epidermidis*	2.5 mg/mL	[61]
2013	Chin et al.		MRSA *A. baumannii* *C. neoformans* VRE *P. aeruginosa* *E. coli* *S. aureus*	0.0039–0.0625 mg/mL	[64]
2015	Cheng et al.		MRSA	4–16 mg/L 2–8 mg/L	[40]
2016	Uppu et al.		MRSA *S. aureus* *E. coli* VREF	0.008–0.25 mg/mL	[70]
2016	Li et al.		MRSA	0.014–0.52 mg/mL	[74]
2019	Yan et al.		MRSA	0.002–0.007 mg/mL	[78]

## Surface-coated polymers containing quaternary ammonium compounds

The importance of coating organo-Si quaternary ammonium chloride (QAC) polymer, a JUC spray on surfaces of medical devices to reduce MRSA contamination in hospital environments, was studied by Yuen et al. in 2015. The bactericidal property is exhibited due to the electrostatic force between the positively charged coated surface and negatively charged cell membrane. The authors demonstrate the antimicrobial coating of QAC polymer in addition to hypochlorite wiping on bed units and high-touch surfaces could significantly reduce the contamination rate in hospital wards [82–85] (Table 2).

**Table 2:** List of polymers containing quaternary ammonium compounds for coating against MRSA

Year	Author	Polymer	Antimicrobial activity	MIC or ZOI	References
2015	Yuen et al.	Organo-Si quaternary ammonium chloride (QAC) polymer	MRSA	Not available	[82]

## Polymers without quaternary ammonium compounds

For hospital infection control, permanent sterile-surface materials were developed by Zhou et al. in 2011. Four guanidine hydrochloride polymers (polyoctamethylene guanidine hydrochloride (POMG) (polymer C_8_), polyhexamethylene guanidine hydrochloride (PHMG) (polymer C_6_), polybutamethylene guanidine hydrochloride (polymer C_4_) and poly(*m*-xylylene methylene guanidine hydrochloride) (polymer C_8(benzene)_)) were synthesized, and their antimicrobial activity was evaluated against meticillin-resistant *S. aureus*, multidrug-resistant *P. aeruginosa*, coagulase-negative staphylococci, ceftazidime-resistant *Citrobacter *spp. and *Enterobacter* spp., vancomycin-resistant *E. faecium*. PHMG and POMG showed immense and tremendous antimicrobial activity against antibiotic-resistant bacteria which causes nosocomial infections. The probable mechanism is that the antimicrobial activity is due to the physicochemical interaction between the bacterial envelop and polymer molecule. The positively charged hydrophobic polymer interacts with the negatively charged phospholipids which damages the cytoplasmic membrane resulting in cell lysis [86–88].

Thoma et al. in 2014 synthesized ammonium ethyl methacrylate homopolymers (AEMPs) with primary ammonium groups in the side chain with various molecular weights (P_7.7_, P_10_, P_12_) by RAFT polymerization. *E. coli*, *P. aeruginosa*, *S. saprohyticus*, *A. baumannii*, *S. aureus*, *Bacillus subtilis*, *Enterococcus faecalis *and MRSA were used to study the antimicrobial activity of the synthesized polymers. These polymers showed a wide range of activity against gram-positive bacteria including MRSA, than gram-negative bacteria. Probably, the primary ammonium groups present in the side chains of the polymer were the cause for disruption of bacterial cell membrane. This article demonstrates that the synthesized polymers could be used for treatment of topical *S. aureus* infections [56, 89, 90].

For the control of hospital-acquired infections, surface-active, photodynamic antimicrobial polymers incorporated with photosensitizers were prepared by McCoy et al. in 2014 and their antimicrobial activity was tested against MRSA and *E. coli*. High-density poly(ethylene) (HDPE) were incorporated with various photosensitizers (TMPyP, TPP, TBO and MB) using hot-melt extrusion process, which exhibits antimicrobial activity in the presence of light. HDPE films and HDPE films incorporated with sentisizers were placed on one another and made into a twin layer by platen press. The reactive oxygen species (ROS) generated due to the irradiation of photosensitizers incorporated in the polymer films reacts with the bacterial cell components (lipids, proteins and nucleic acids) and causes cell death. HDPE films incorporated with TMPyP exhibited excellent antimicrobial activity against MRSA in the presence of light [91–93].

Labena et al. in 2016 synthesized hyperbranched poly(amidoamine) (h-PAMAM) with various terminal groups (h-PAMAM-ester, h-PAMAM-amine, h-PAMAM-amine plus) by repeated Michael addition and amidation to enhance reliability of the synthesis. The antimicrobial activity was tested against *B. subtilis*, *Candia albicans*, *S. aureus*, *Aspergillus niger*, *P. aeruginosa*, *E. coli* and MRSA. The electrostatic interactions between the cell membrane and h-PAMAM molecules cause denaturation of membrane’s protein and enter into phospholipid layer, which with raise in permeability causes membrane destabilization followed by intracellular structure leakage which leads to bacterial cell death. The authors demonstrated that h-PAMAM with amine terminations (amine and amine plus) showed broad-spectrum antimicrobial activity against MRSA [94–97].

A series of amphiphilic, cationic polycarbonate polymers containing primary amino groups (single, diblock and random) was synthesized by Nimmagadda et al. in 2016, and their antimicrobial activity was tested against three gram-positive bacterial strains vancomycin-resistant *E. faecalis* (VREF), methicillin-resistant *S. aureus* (MRSA) and methicillin-resistant *S. epidermidis* (MRSE). The random polymers exhibited a wide range of antimicrobial activity than single or diblock polymers. The random polymer with 20 hydrophobic and 20 hydrophilic units showed excellent activity against multidrug-resistant bacteria (MRSA). The polymer micelle on contact with bacterial surface breaks into small entities due to change in electrostatic interactions. Due to the amphipathic nature of the free polymer chain, it enters through the surface of bacteria which disrupts the bacterial membrane and leads to cell death; the mechanism was studied by TEM [65, 66, 98, 99].

A series of six cationic chitosan derivatives, *N*-(2-hydroxypropyl)-3-trimethylammonium chitosan chlorides (HTCC), by changing the mole ratio of glycidyltrimethylammonium chloride (GTMAC) were synthesized by Hoque et al. in 2016. The synthesized cationic polymers on interaction with anionic bacterial membrane disrupt the cell membrane leading to cell death. The mechanism of action was confirmed by several microscopic and spectroscopic methods. The antifungal and antibacterial property of the prepared HTCC polymers was evaluated against drug-sensitive bacteria (*A. baumannii*, *S. aureus* and *E. coli*) and MDR bacteria (VRE, MRSA and β-lactam-resistant *K. pneumonia*). Among the synthesized series of derivatives, two active polymers (HTCC3 and HTCC6) showed significant activity against MRSA in a murine model of superficial skin infection [100–103].

In another study, Kamaruzzaman et al. in 2016 found that a cationic polymer, polyhexamethylene biguanide (PHMB), had excellent antimicrobial properties and could be treated against intracellular MRSA (EMRSA-15 and USA 300). PHMB co-localizes with intracellular MRSA in keratinocytes, indicating that killing occurs by direct interactions inside host cells. The authors conclude that PHMB has potential to treat skin infections caused by intracellular MRSA and other intracellular bacteria [36, 104–106].

Hong et al. in 2017 synthesized cationic amphiphilic random methacrylate copolymer (PE_31_) with pH-responsive activity by RAFT polymerization. The mechanism explained is electrostatic interaction occurs between the positively charged polymer and negatively charged cell wall. On interaction, the hydrophobic side chains of a polymer enter into hydrophobic portion of bacterial lipid membrane, which causes disruption of membrane leading to bacterial cell death. The authors concluded that the polymer showed high antimicrobial activity against vancomycin-intermediate *S. aureus* and methicillin-resistant *S. aureus* at neutral pH [107–110].

Biocompatible, inexpensive, water-soluble macromolecular antimicrobial polyionenes were developed by Liu et al. in 2017 for the treatment of hospital-acquired and MDR infections. Catalytic-free, polyaddition polymerization process was used for the synthesis of series of antimicrobial polyionenes. Hydrophilic and hydrophobic groups were distributed alternatively in the polymer chain, among which hydrophobic components were in contact with negatively charged cell wall. The hydrophobic components target the lipid bilayer causing cytoplasmic membrane disruption and resulting in cell lysis. The antimicrobial activity of synthesized polymers was examined against MRSA, *S. aureus*, *K. pneumonia*, *A. bacumanii*, *C. neoformans*, *E. coli* and *C. albicans* [59, 111, 112].

An antimicrobial polymer which showed activity against *S. aureus*, MRSA, MSSA and other HAIs was synthesized by Mercer et al. in 2017. The authors prepared NP 108, a cationic, poly-lysine polymer made up of amino acid building blocks which was water-soluble and had a wide range of antimicrobial activity. The macromolecule shows membrane-acting bactericidal activity due to net positive charge present on the polylysine, which causes disruption of cell membrane resulting in cell death. These polymers were used for nasal delcolonization of *S. aureus* and prevention of HAI [53, 113, 114].

To eradicate multidrug-resistant (MDR) bacterial infections, Chin et al. in 2018 synthesized a biodegradable macromolecule, guanidinium-functionalized polycarbonates. The synthesized polymers (pEt_10 and pEt_20) showed broad-spectrum antimicrobial activity against MDR *P. aeruginosa*, *E. coli*, *A. baumannii*, MRSA and *K. pneumonia.* This polymer also exhibits electrostatic interaction between the polymer and cell wall, and targets cytoplasmic membrane resulting in releasing of cell constituents which leads to cell death. These polymers were less toxic and had great potential for the treatment and prevention of MDR systemic infections [115–118].

Poly(*para*-phenylene ethynylene) (PPE)-and poly(*para*-phenylene vinylene) (PPV)-poly[(2-(methacryloyloxy)ethyl)trimethylammonium chloride] (PMETAC) graft copolymers of low and high molecular weights were synthesized by Damavandi et al. in 2018. The cationic side chains present in the conjugated polymers interact with the negatively charged surface of bacterial cell and exhibit antimicrobial activity. The antimicrobial activities of synthesized polymers were tested against MRSA, *E. coli*, *E. faecium* and *A. baumannii.* The authors found that the low molecular weight PPE-g-PMETAC copolymer showed significant antimicrobial activity [119–121].

In 2019, Hong et al., studied about novel antimicrobial polyionene, poly(*N*,*N*′-(ethane-1,2-diyl)*bis*(4-(chloromethyl)benzamide)-*co*-tetramethyl-1,3-diaminopropane), synthesized by Lou et al. in 2018 for the treatment of MRSA-induced bloodstream infection. Antimicrobial polymers form pores on plasma which leads to leakage of cytoplasmic components resulting in cell death. The authors demonstrated that the polymer possessed strong antimicrobial activity against MRSA. Due to negligible toxicity and potential therapeutic effect, the polymer could be used to treat MRSA caused, especially blood stream infections [31, 122, 123].

Electrospun fiber mats were synthesized by Boncu et al. in 2019 for the treatment methicillin-resistant *S. aureus* (MRSA) associated with bone infections and soft tissues. Biodegradable polymers, poly(lactic-*co*-glycolic) acid (PLGA) and polycaprolactone (PCL) loaded with linezolid, were used for preparing electrospun fiber mats. The antibacterial activity of synthesized fiber mats was examined against isolated bacteria (MRSA) which causes prosthetic infections. The fibers were non-toxic, biocompatible, biodegradable and had long-term activity [124–126].

Kuroki et al. in 2019 synthesized series of ammonium and guanidinium polymers of various sequences (statistical, diblock and tetrablock) by RAFT polymerization. The synthesized polymers were tested for antimicrobial activity against MDR methicillin-sensitive strains (MSSA) and methicillin-resistant *S. aureus* (MRSA). The authors concluded that diblock guanidinium (GD30) polymer had major impact for the treatment of intracellular, MDR bacteria (MSSA and MRSA). Here bacterial DNA binding and pore formation both would have occurred, leading to death of bacteria [127–130].

For the treatment of MRSA a new method, Antibacterial Photodynamic Therapy (APDT) was used by Guo et al. in 2020, to deliver photosensitizers. In this article self-assembled, lipase sensitive micelle was developed to deliver hydrophobic hypocrellin A (HA). Polymeric micelle made up of methoxy poly(ethylene glycol)-block-poly(ε-caprolactone) (mPEG-PCL/HA), an amphiphilic copolymer, was used to encapsulate HA. The polymeric micelles could release HA in the presence of lipase, on irradiation of light or in appropriate wavelength range and this improved the APDT activity. These mPEG-PCL/HA micelles showed high activity against MRSA and could be used to combat MRSA infections [131–134].

Christofferson et al. in 2020 synthesized diblock and triblock oligomers by photo-induced atom transfer radical polymerization and studied their antimicrobial activity against gram-positive bacteria namely *S. aureus* and MRSA. In this article, it has been reported that the triblock oligomers showed excellent antibacterial activity ~ 99% and 98% against *S. aureus* and MRSA compared to the diblock oligomers, because the oligomer systems had conformational differences. The interaction between peptidoglycan functional group leads to the disruption of peptidoglycan layers [135, 136] (Table 3).

**Table 3:** List of polymers synthesized with various functional groups against MRSA and other pathogenic microorganisms

Year	Author	Polymer	Antimicrobial activity	MIC or ZOI	References
2011	Zhou et al.		MRSA, multidrug-resistant *P. aeruginosa*, coagulase-negative staphylococci, ceftazidime resistant-*Citrobacter *spp. and -*Enterobacter *spp., vancomycin resistant *E. faecium*	0.001–0.008 mg/mL	[86]
2014	Thoma et al.		MRSA *E. coli* *S. aureus* *B. subtilis* *E. faecalis* *P. aeruginosa* *S. saprohyticus* *A. baumannii*	P_7.7_-125 mg/mL P_10_-83 mg/mL P_12_-63 mg/mL	[56]
2014	McCoy et al.	High-density poly(ethylene) (HDPE)	MRSA *E. coli*	Not available	[91]
2016	Labena et al.		MRSA *B. subtilis* *P. aeruginosa* *E. coli* *S. aureus* *A. niger* *C. albicans*	0 (ester) 0.0078 mg/mL (amine) 0.013 ± 0.0037 mg/mL (amine plus)	[94]
2016	Nimmagadda et al.		MRSA MSSA VREF	0.0016 mg/mL	[98]
2016	Hoque et al.		MRSA VRE β-lactum *K. pneumonia* *A. baumannii* *S. aureus* *E. coli*	0.125–0.250 mg/mL	[100]
2016	Kamaruzzaman et al.		MRSA	0.004 mg/mL	[36]
2017	Hong et al.		MRSA VISA	0.015–0.200 mg/mL	[107]
2017	Liu et al.		MRSA *S. aureus* *K. pneumonia* *A. bacumanii* *C. neoformans* *E. coli* *C. albicans*	0.00195–0.0313 mg/mL	[111]
2017	Mercer et al.	NP108 (poly-lysine)	MRSA MSSA *S. aureus*	0.00025–0.0005 mg/mL	[53]
2018	Chin et al.		MRSA *P. aeruginosa* *E. coli* *A. baumannii* *K. pneumonia*	0.008–0.016 mg/mL	[115]
2018	Damavadi et al.		MRSA *E. coli* *E. faecium* *A. baumannii*	0.00001 mg/mL	[119]
2019	Hong et al.		MRSA	0.004 mg/mL	[31, 122]
2019	Boncu et al.	Poly(lactic-*co*-glycolic) acid (PLGA) and polycaprolactone (PCL) loaded with linezolid	MRSA	21–42 mm	[124]
2020	Guo et al.	m PEG-PCL/HA micelle	MRSA	0.00069 mg/mL	[131]
2020	Christofferson et al.,		*S. aureus* MRSA	~ 98%	[135]

## Surface-coated polymers without quaternary ammonium compounds

Antifouling surface coatings were developed with antimicrobial properties on silicon rubber to fight against intravascular catheter-associated infections (CAIs) using diblock copolymers which was synthesized by Ding et al. in 2012. (PEG-*b-*cationic polycarbonates) were synthesized by metal-free organocatalytic ring-opening copolymerization of poly(ethylene glycol) (PEG) and cationic polycarbonate. Polymers coatings were developed using reactive polydopamine (PDA) to increase antimicrobial properties of substrate surface. These properties of the polymeric coatings were systematically investigated against methicillin-resistant *S. aureus* and methicillin-susceptible *S. aureus*, which are the major causes of intravascular catheter-associated infections. The tests were carried out for various polymers compositions. The hydrophobic monomer unit present in the polymer may interact with the bacterial cell membrane by incorporating into the lipid domain and resulting in cell death [137–140].

The efficacy of biodegradable poly-d,l-(lactide) (PDLLA) polymer solution loaded with linezolid antibiotic and coated on orthopedic Kirschner wires (K-wires) by dip coating technique to prevent the adhesion of MRSA was studied by Kaur et al. in 2014. The adherence of MRSA was evaluated on naked wires, PDLLA wires, K1, K2 and K3 wires (PDLLA impregnated with three different concentrations of linezolid (2.5%, 5% and 10%)). The authors concluded that K2 and K3 wires decreased bacterial adhesion by 60% when compared to K1 (which decreased by 40%), PDLLA and naked wires. The reduction in the bacterial attachment on wires was correlated with the amount of drug released from the wires [141–144].

Dinjaski et al. in 2014 studied the antimicrobial properties of poly-3-hydroxy-acetyllthioalkanoate-*co*-3-hydroxyalkanoate (PHACOS) containing thioester groups in the side chains by comparing it with non-reactive poly(3-hydroxyoctanoate-*co*-hydroxyhexanoate) (PHO) and poly(ethylene terephthalate) (PET). PET disks were coated with PHACOS and PHO by solvent casting, and uncoated PET disks were used as control for examining the antimicrobial activity and bacterial adhesion of *S. aureus* subsp. *aureus*, *Streptococcus pyogenes*, *Mycobacterium smegmatis*, *B. subtilis *subsp.* subtilis*, *E. coli*, *S. epidermidis*, MRSA, *P. aeruginosa* and *Streptococcus dysgalactiae *subsp. *equisimilis*. The bacterial activity of *S. aureus* on PHO was more compared to that of PHACOS, which demonstrates that PHACOS possesses anti-staphylococcal activity. In addition to this, PHACOS effectively inhibits the growth of MRSA. This activity is exhibited by functionalized side chains which possess thioester groups. This article concludes that PHACOS acts as contact active surface which decreases the adhesion of *S. aureus* and MRSA, and hence can be used in biomedical implants as an infection-resistant material [110, 145–147].

Polyastaxanthin (p (ATX)) coatings were developed by Weintraub et al. in 2018, and their antimicrobial activity was examined against *S. aureus* (MRSA and MSSA) and *S. epedermidis* by coating them on polyurethane catheters. Since the polymeric coating material was biodegradable and had excellent antimicrobial properties, authors concluded that the coatings could be used as antimicrobial coating for medical devices [148–150] (Table 4).

**Table 4:** List of polymers synthesized with various functional groups for coating against MRSA and other pathogenic microorganisms

Year	Author	Polymer	Antimicrobial activity	MIC or ZOI	References
2012	Ding et al.		MRSA MSSA	1.88 mm	[137]
2014	Kaur et al.	Poly-d,l-(lactide) (PDLLA) polymer	MRSA	0.002–0.004 mg/mL	[141]
2014	Dinjaski et al.		*MRSA* *S. aureus *subsp. *Aureus* *S. pyogenes* *M. smegmatis* *B. subtilis* subsp. *Subtilis* *E. coli* *S. epidermidis* *P. aeruginosa* *S. dysgalactiae* subsp. *equisimilis*	Not Available	[145]
2018	Weintraub et al.		MRSA MSSA *S. epidermis*	Not available	[148]

The antimicrobial activity exhibited by the polymers depends on the molecular weight [56, 64, 100, 119, 135], alkyl chain length [64, 70, 86, 115] and terminal functional group [94] of the polymers. The synthesized polymers had various advantages such as biocompatibility and low toxicity and thus were used for several applications such as in biomedical implants, disinfectants for hospital infection control, in orthopedic operations, treatment of nasal colonization infections, skin infections and bloodstream infections. Few polymers were used for coating kirschner wires, polyurethane tubes, etc., to prevent the spread of bacteria. Certain polymers had resistant ability only toward few pathogens, and they had MIC values slightly higher compared to their corresponding standards due to which the application was limited in biomedical field.

## Conclusion

Drug-resistant MRSA which causes numerous deadly infectious diseases, namely tympanitis, pneumonia, purulent meningitis pleuritis and bloodstream infection, can be reduced by the approach toward polymeric synthesis. This article reports the polymers synthesized by various research groups to treat MRSA since last decade. Polymers with various beneficial properties like biocompatibility, stability (thermal and mechanical) and antimicrobial activity were synthesized, and their ability to prevent MRSA has been described. This article also discusses the authors who showed interest in loading antibiotics to polymeric backbones to fight against MRSA. On the whole, the mechanisms explained for various polymers are in two ways (i) electrostatic interaction between polymers and cell wall and (ii) polymers targeting cytoplasmic membrane. Future research in the field of combating MRSA can be focused on polymer surface coatings to restrain the spread of MRSA and other multidrug-resistant bacteria.
